# What is the role of heat shock protein in abdominal organ transplantation?

**DOI:** 10.31744/einstein_journal/2022RB6181

**Published:** 2022-03-04

**Authors:** Igor Lepski Calil, Francisco Tustumi, Jorge Henrique Bento de Sousa, Bruno Martins Tomazini, Ruy Jorge Cruz, Gustavo Niankowski Saliba, Rafael Antonio Arruda Pécora, Luiz Augusto Carneiro D’Albuquerque

**Affiliations:** 1 Hospital Israelita Albert Einstein São Paulo SP Brazil Hospital Israelita Albert Einstein, São Paulo, SP, Brazil.; 2 Faculdade de Medicina Universidade de São Paulo São Paulo SP Brazil Faculdade de Medicina, Universidade de São Paulo, São Paulo, SP, Brazil.; 3 Hospital Sírio-Libanês São Paulo SP Brazil Hospital Sírio-Libanês, São Paulo, SP, Brazil.; 4 School of Medicine University of Pittsburgh Pittsburgh PA USA School of Medicine, University of Pittsburgh, Pittsburgh, PA, USA.

**Keywords:** Reperfusion injury, Heat-shock proteins, Kidney transplantation, Liver transplantation, Organ transplantation

## Abstract

Ischemia-reperfusion injury is a pathophysiological event occuring after abdominal organ transplantation, and has a significant influence on prognosis and survival of the graft. It is involved in delaying the primary function or non-functioning of the graft. The objective of this study was to provide information on heat shock protein mechanisms in ischemia-reperfusion injuries in abdominal organ transplantations, and to indicate the possible factors involved that may influence the graft outcome. Several classes of heat shock proteins are part of the ischemia and reperfusion process, both as inflammatory agonists and in protecting the process. Studies involving heat shock proteins enhance knowledge on ischemia-reperfusion injury mitigation processes and the mechanisms involved in the survival of abdominal grafts, and open space to support therapeutic future clinical studies, minimizing ischemia and reperfusion injuries in abdominal organ transplantations. Expression of heat shock proteins is associated with inflammatory manifestations and ischemia-reperfusion injuries in abdominal organ transplantations and may influence graft outcomes.

## INTRODUCTION

### Heat shock proteins

Heat shock proteins (HSP) are part of a large family of proteins and are found in virtually all organs and tissues.^(1)^ Heat shock proteins are essential in a wide variety of intracellular processes, such as protein folding, assembly, disassembly and intracellular localization; and secretion, regulation and degradation of other proteins.^(2,3)^They have an important protective role, both under stress-free conditions,^(4)^ and during ischemic or oxidative stress, inflammatory processes, intracellular energy depletion, hormonal processes, apoptosis, among others.^(2,5)^These stress factors occur in patients undergoing abdominal organ transplantation, and the ability to withstand such stressors is closely linked to the success of the procedure.

When the blood supply to a tissue is interrupted, a sequence of biochemical events is initiated, leading to cell dysfunction and death. Restoring blood flow is a prerequisite for recovery from ischemic injury. However, reperfusion can induce more local tissue damage.^(6)^ This injury is at the pathophysiological basis of several serious diseases, including myocardial infarction, stroke, tissue damage induced by surgical procedures and organ transplantation.^(7)^ Understanding HSPs could contribute to the development of clinical interventions capable of reducing the extent of ischemia-reperfusion (I/R) injury, such as in organ transplantations.

Ischemia-reperfusion injury is a pathophysiological event that occurs after abdominal organ transplantation and has a significant influence on the prognosis of graft function. The mechanisms of this injury remain unclear. Several factors are associated with the I/R injury process, including anaerobic metabolism, oxidative stress, intracellular calcium overload, innate immune responses, activated cytokines and chemokines.^(8)^

In abdominal organ transplantation, I/R is essential for graft survival and is involved in different injuries, such as late graft function and primary non-function of the graft. The discrepancy between supply and demand for donors has led to an increase in transplantation of marginal organs, such as extended criteria donors, and donation after cardiac death.^(9)^ Such marginal organs are more susceptible to I/R injury.^(10-12)^

Heat shock proteins are described as intracellular chaperones of aberrant denatured, non-native and denatured proteins, and are involved in cytoprotection and adaptation for cell survival in response to stressful stimuli. Under more intense stress, HSPs are released into the extracellular environment, as cytokines, and can be called chaperokines. The chaperokine activity of HSPs is mediated, in part, by the interaction with toll-type receptors, leading to the activation of different immunological pathways, such as acute rejection, chronic rejection, and I/R injury.^(13-17)^

The objective of this review was to provide information on the mechanisms of HSP in I/R injury in abdominal organ transplantations, and to indicate the possible factors involved that may influence the graft outcome. Studies involving HSPs allow for greater knowledge on the processes controlling attenuation of I/R injuries and the mechanisms involved in the survival of abdominal grafts, and open space to support future clinical therapeutic studies, minimizing I/R injuries in abdominal organ transplantations.

This is a narrative review of the literature, evaluating the mechanisms involved in HSP in I/R injuries in abdominal organ transplantations. The databases consulted were PubMed^®^, Latin American and Caribbean Literature in Health Sciences (LILACS), and Embase. The keywords searched were “*ischemia*”, “*transplantation*”, “*reperfusion injury*”, “*nitric oxide*”,“*heat-shock protein*”, “*HSP*”, “*liver*”, “*kidney*”, “*pancreas*”, “*islets of Langerhans*”, “*intestine*” and “*bowel*”. All references were reviewed to retrieve additional articles.

### Historical aspects

Researching the salivary glands of drosophilae, Ritossa described, for the first time, in 1962, a group of proteins, whose expression was modified with temperature shocks. Such proteins would be called “heat shock proteins”.^(18)^

Later, it was discovered that, in addition to temperature shock, several stressful situations could induce such alterations in the chromosomal areas associated with the synthesis of HSP. Ritossa demonstrated that 2,4-dinitrophenol could induce an increase in HSP synthesis.^(18)^ In 1976, Koninkx, in addition to 2,4-dinitrophenol, investigated arsenite, vitamin B6, and anaerobiosis and demonstrated all these stressors could induce an increase in the HSP synthesis rate.^(19)^

Since then, the scientific community had great interest in investigating the role of such proteins in the response to stress situations.

## FAMILIES OF HEAT SHOCK PROTEINS

Heat shock proteins are usually named according to the size of their monomers.^(20)^ They can be grouped into the following protein families:^(1)^ HSP110, HSP90, HSP70, HSP40, small HSPs, and human chaperonin families (Table 1). The different families of HSPs work together.

**Table 1:** Heat shock protein families

Family	Cellular location	Function
HSP90	Cytosol, nucleus, endoplasmic reticulum, and mitochondria	Signal transduction; cell proliferation; protein folding
HSP70 and HSP110	Cytosol, nucleus, endoplasmic reticulum, and mitochondria	Anti-apoptotic activity; signal transduction; cell proliferation; intracellular transport
Human chaperonines	Mitochondria and cytosol	Protein stability; protein folding
HSP40	Nucleus	Cofactor for HSP70 ATPase activity
Small HSPs	Cytosol	Anti-apoptotic activity; actin filament stabilization

HSP: heat shock proteins.

## ROLE OF HEAT SHOCK PROTEINS IN ORGAN TRANSPLANTATION

The stressful environment caused by I/R injuries in transplantations stimulates the synthesis of HSPs, which would have a protective role on transplanted organs.^(13-17)^ In studies on dynamic organ preservation methods, Minor et al. and von Horn et al.^(21,22)^ demonstrated that short hyperthermic impulses could induce HSP synthesis, which, in turn, causes a reduction in the degradation of mitochondrial enzymes and an increase in bile production after reperfusion.

Therefore, HSPs could be considered prognostic biomarkers in organ transplantations. The mapping of I/R-related biomarkers points to possible organ conditioning during normal or hyperthermic dynamic preservation methods.^(23,24)^

### Intestinal transplantation

Intestinal transplantation is an accepted treatment for patients with irreversible and complicated bowel failure. However, it is characterized by higher acute and severe rejection rates among abdominal organ transplantations.^(25)^ Through the interaction of innate and adaptive immunological systems, I/R injury exacerbates the alloimmune response.^(26-28)^

Acute rejection is the most important factor in determining the survival of intestinal allografts.^(29)^ Ogita et al.^(29)^ evaluated the expression of HSP60 and HSP70 in the graft and in the native bowel after heterotopic small bowel transplantation in rats. They demonstrated that HSP expression is induced after small bowel transplantation, and its induction has both immunological and non-immunological components. They also showed that HSPs are induced in the native bowel, and expression in the graft and in the recipient bowel is only partially inhibited by the administration of tacrolimus immunosuppression.

In animal experiments, Oltean et al.^(30)^ observed a direct relation between the expression of HSP60 and immune activation, which reflects in the degree of rejection. The hypothesis that the increase in HSP60 is due to immunological causes is consistent with the observation that the increase in HSP60 was seen only in the allogeneic combination. Furthermore, the increase was clear in the crypt area, the same place where rejection becomes more exacerbated. Oxidative stress and inflammation are the driving forces of intestinal I/R injury.^(12)^ Consequently, intestinal I/R is also expected to activate anti-inflammatory pathways, such as HSPs, to regulate the inflammatory response to injury.^(31)^

In an experimental model, Vincenti et al.^(32)^ demonstrated that HSP32 and HSP70 inflammatory response is similar when an arterial clamping is performed, compared to when a venous occlusion is performed.

### Liver transplantation

When stimulated by environmental stress factors, cells expressing HSP70 can survive even under conditions that would normally be lethal to them.^(33)^ Thus, HSP70 can be a potential prognostic biomarker in liver transplantation. Animal models have shown that liver preconditioning, such as by transient clamping of the hepatic pedicle, (Pringle maneuver), can induce increased expression of HSP70, thus leading to tolerance to I/R lesions.^(34,35)^

Conti et al.^(36)^ evaluated gene expression levels in reperfused livers compared to baseline values before the removal of donor organs. They observed that 795 genes had their expression modified by the I/R lesion, wherein 12.5% of genes were related to the inflammatory process and 12% to apoptotic process. The genes that encode HSPs accounted for 22.5% of total set of genes that had their expression modified. The most regulated genes were HSP70, HSP105, and HSP90.

Different experimental models in rats have shown that preconditioning influences the production of HSP, especially HSP72 and HSP90, through controlled hyperthermia and I/R obtained with the Pringle maneuver or transplantation. In these studies, the intervention group had a significantly greater increase in survival, lower values of released liver enzymes, and less histological liver inflammation and hepatocellular necrosis.^(37,38)^

Chen et al.^(39)^ showed that mice with overexpression of HSP27 are protected against I/R. The protective mechanism involves reduced necrosis and apoptosis of the liver parenchyma, better preservation of the vascular barrier function, and less neutrophil infiltration. Wolf et al.^(40)^ hypothesized that HSP70 plays an important role in liver regeneration after hepatectomy, by performing partial hepatectomy in mice without HSP70. These rats had decreased liver regeneration, and the hypothetical mechanism was that HSP70 contributes to early liver regeneration via potentiation of the expression of tumor necrosis factor-alpha (TNF-α). Yang et al.^(41)^ demonstrated that the levels of HSP70 and HSP27 were increased in both the I/R and I/R groups associated with octreotide, but were especially higher in the latter group. These findings show a possible therapeutic role for octreotide through the upregulation of HSP27 and HSP70.

Knowing that geranylacetone has HSP-inducing properties, Fudaba et al.^(42)^ analyzed preconditioning with geranylacetone in donor rats after orthotopic liver transplantation. When rats were pre-treated with geranylacetone, an increase in HSP72 and HSP90 was observed after ischemic stress, which was not observed in the absence of ischemia, indicating that geranylacetone acts synergistically with ischemic stress in the liver in inducing the production of HSP, reduces I/R and improves the survival rate.

### Kidney transplantation

The intracellular forms of HSP, especially HSP70, have a cytoprotective action and play a role in delaying apoptosis.^(43)^ After cell injury, HSP70s repair damaged proteins and mark those that are not repairable for subsequent removal, preventing the accumulation of these affected tissues.^(44)^

In this way, HSP70 has the ability to maintain cell structure stable and enable cell survival. Heat shock proteins protects mitochondria from oxygen free radicals, maintains stable intracellular levels of adenosine triphosphate (ATP), and prevents excessive calcium influx, which would cause their destruction.

In experimental models of I/R kidney injury, HSPs (and particularly HSP70) play a crucial role in restoring the cellular polarity of renal tubular cells and repairing essential proteins, which are involved in stabilizing cytoskeletal structures. In the context of a post-renal transplantation patient, HSP seems to play an even more important role. In this case, insults mediated mainly by immunological factors lead to an imbalance between pro- and anti-oxidant substances, whose final route is tubular interstitial fibrosis, glomerulosclerosis, and vascular destruction.^(45)^

Some studies have shown an important role for HSP27 in this population. High concentrations of HSP27 are present in the medulla, the main renal region to suffer ischemic alterations. HSP27 seems to have the ability to modulate the proliferation of actin filaments, preventing fibrous proliferation in vascular musculature and mesangial cells.^(46)^

### Pancreatic transplantation

With current advances in surgical techniques and immunomodulation strategy, survival after pancreatic transplantation has increased in the last two decades. However, I/R injury resulting from pancreatitis remains a major therapeutic challenge, with an incidence in the range of 17% to 35% of these patients.^(47)^

Tumor necrosis factor-alpha is important in cell proliferation and differentiation, as well as in apoptosis and necrosis related to inflammatory processes. Some studies have demonstrated *in vitro* I/R injury mediated by TNF-α secreted by pancreatic acinar cells.^(48)^ This inappropriate secretion results in activation of local inflammatory response, with changes in microcirculation, which perpetuate the cycle of local injury. HSP70 regulates excessive TNF-α secretion, reducing local inflammation.

In a study in pigs undergoing pancreatic islet cell transplantation, the amount of HSP70 rapidly increased, until 3 to 9 days after the transplantation, when the levels of HSP70 were reduced again.^(49)^

Also in animal models, increased expression of HSP70 artificially induced by application of glutamine attenuates the lesion induced by interleukin 1 beta (IL-1β) in association with the production of nitric oxide (NO), modulating ischemic lesions in islet transplantation.^(50)^

## CONCLUSION

Expression of heat shock protein is associated with inflammatory manifestations and ischemia-reperfusion injury in abdominal organ transplantations. This is a prognostic factor and can influence the graft outcome.
